# Halos and Hemoptysis: A Case of Intrapulmonary Sequestration Masquerading as a Pulmonary Aspergilloma

**DOI:** 10.1155/crpu/4242228

**Published:** 2026-03-08

**Authors:** Faisal Masood, Anjana Murali, Claire Fishman

**Affiliations:** ^1^ Internal Medicine, University of Pennsylvania Perelman School of Medicine, Hospital of the University of Pennsylvania, Philadelphia, Pennsylvania, USA, pennmedicine.org

## Abstract

**Introduction:**

Bronchopulmonary sequestration (BPS) is a rare congenital malformation of the lung characterized by nonfunctional pulmonary tissue without communication to the tracheobronchial tree and with anomalous arterial supply. Intralobar sequestration (ILS), the more common form, typically presents in childhood or early adulthood.

**Case Presentation:**

We describe the case of an 84‐year‐old woman with a history of untreated rheumatoid arthritis, coronary artery disease, and remote smoking, who presented with recurrent hemoptysis over 6 months. Initial evaluation revealed lower lobe cystic changes with an internal solid component and diffuse ground‐glass opacities on chest CT, raising suspicion for fungal etiology. Serial imaging demonstrated interval progression of the cystic lesion with an enlarging solid component. The patient was treated with empiric oral antifungal therapy, but persistent hemoptysis and radiographic changes prompted surgical consultation. A left lower lobe segmentectomy was performed for definitive management. Histopathology revealed abnormal pulmonary parenchyma with dilated airspaces, mucus‐filled airways, and enlarged vessels, consistent with intralobar BPS. No fungal elements or microorganisms were identified. This case appears to represent the oldest reported age at diagnosis of ILS.

**Conclusion:**

This case underscores the importance of maintaining a broad differential diagnosis for recurrent hemoptysis in elderly patients. While infection, malignancy, and autoimmune disease are common considerations, congenital anomalies such as ILS can rarely present late in life. Awareness of this possibility may help avoid diagnostic anchoring and guide appropriate multidisciplinary management.

## 1. Introduction

Hemoptysis is a clinically significant symptom with a broad differential diagnosis ranging from infection and malignancy to autoimmune and vascular disease. While mild in volume, even recurrent small episodes warrant thorough evaluation, as hemoptysis can reflect underlying pathology with potentially life‐threatening implications. Cross‐sectional imaging and bronchoscopic evaluation often assist in diagnosis, though the cause may remain elusive in a subset of patients.

Bronchopulmonary sequestration (BPS) is a rare congenital anomaly characterized by nonfunctional lung tissue with aberrant systemic blood supply and no communication with the tracheobronchial tree [1]. Intralobar sequestration (ILS), the more common variant, typically presents in childhood or young adulthood with recurrent infections or respiratory symptoms [1]. Diagnosis in elderly patients is exceptionally uncommon, and when present, ILS may mimic more prevalent conditions such as fungal infection or interstitial lung disease.

## 2. Case Presentation

**Figure 1:**
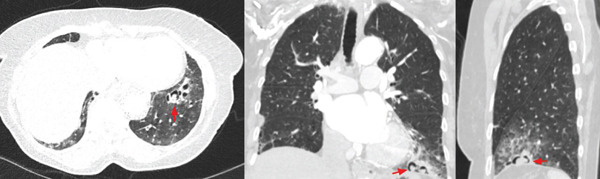
CT chest with diffuse, lower lobe predominant regions of subpleural cystic change, which may be manifestations of rheumatoid‐related or other interstitial lung disease. More focal regions of cystic change visualized at the left lung base with internal solid component (arrow) are seen in axial, coronal, and sagittal cross sections.

An 84‐year‐old woman with history of untreated rheumatoid arthritis, 20 pack‐year smoking history, CAD, and a descending penetrating aortic ulcer s/p repair presented with recurrent episodes of hemoptysis over 6 months. Apart from small‐volume hemoptysis every several weeks, the patient denied persistent cough, sputum production, fevers, weight loss, dyspnea, chest pain, or any change to her functional status. She was overall unconcerned, but as her third instance of hemoptysis was approximately 1–2 oz, she presented for evaluation. Initial ER visits and hospitalization revealed lower lobe cystic changes and ground‐glass opacities on CT chest, raising concerns for a chronic fungal infection (Figure 1). Despite negative aspergillus IgG and no clinical improvement with empiric bacterial pneumonia treatment, the characteristic cystic change at the left lung base with an internal solid component sustained high suspicion for fungal infection. With no further hemoptysis during her inpatient stay, she opted for discharge and outpatient surveillance with serial lung CTs.

Weeks after discharge, the patient experienced another episode of 2‐oz hemoptysis, prompting readmission. Overall, the patient was well‐appearing and continued to deny any chest pain, dyspnea, fevers, or other signs of systemic infection. Objective information was also reassuring as the patient′s hemoglobin and platelets remained stable from her prior ED encounter; her physical exam was largely unrevealing. She underwent a repeat CT chest with and without contrast: There was no evidence of large volume active bleeding in the thorax; however, compared to her prior CT weeks prior, there was interval progression noted with increased solid component within cystic spaces in a lung base. Otherwise, there was similar appearance of the lung parenchyma including diffuse ground‐glass attenuation, tree‐in‐bud opacities in the lung bases, and multifocal pulmonary nodules with potential differentials including but not limited to rheumatoid lung nodules, mycotic infection, sarcoidosis, and neoplastic etiologies.

Given recurrent hemoptysis and progressive imaging findings, she was started on empiric PO posaconazole. While the patient remained stable, the recurrence of her hemoptysis and readmission prompted further investigation and definitive management; interventional radiology (IR) was consulted for potential intracavitary amphotericin injection for empiric antifungal treatment versus bronchial artery embolization (BAE) for symptom control. However, after further discussions and shared decision‐making, the team opted for a surgical consultation for pathological confirmation, leading to a left lower lobe segmentectomy. Surprisingly, surgical pathology revealed no fungal infection but rather noted distinct transitions to an area of abnormal lung parenchyma with enlarged airspaces and dilated airways. Airways were noted to be mucus‐ and blood‐filled with adjacent enlarged vessels. The cystic area was extensively sectioned, and no evidence of a fungal ball was identified; extensive staining was negative for microorganisms. Overall, the histologic appearance of the patient′s lung parenchyma was most consistent with a diagnosis of ILS, a rare congenital anomaly where nonfunctional pulmonary tissue develops separately from the functional tracheobronchial tree and may present as hemoptysis in older adults [1]. A brief comparison of the differential diagnosis of lung pathology in this case is outlined in Table 1.

**Table 1:** A comparison of aspergillomas, ILS, and RA‐related lung disease.

	Aspergilloma	Intralobar sequestration (ILS)	Rheumatoid arthritis (RA)–related lung disease
Epidemiology	Patients with pre‐existing lung cavities (i.e., tuberculosis, sarcoidosis, and bronchiectasis)	Congenital anomaly	Patients with RA (10% have clinical interstitial lung disease, 30%–60% have radiographic lung disease)
Typical age group	Adults, usually middle‐aged, with history of chronic lung disease	Often diagnosed in adolescents though can be made in infancy or on prenatal ultrasound	Middle‐aged to older adults with RA
Serology	Aspergillus IgG	N/A	Rheumatoid factor, anticyclic citrullinated peptide
Imaging findings	‐ Mobile intracavitary mass (“Monod sign”) with air crescent sign‐ Surrounding pleural thickening‐ Fungal ball movement with positional changes	‐ Mass‐like opacity predominantly in lower lobes‐ Cystic changes or bronchiectasis within lesion‐ Recurrent pneumonia in the same location	‐ Usual interstitial pneumonia (UIP) or nonspecific interstitial pneumonia (NSIP) pattern‐ Rheumatoid nodules (i.e., multiple peripheral nodules that may cavitate)
Vascular supply	Normal pulmonary vasculature	Systemic arterial supply (typically thoracic aorta) with venous drainage into pulmonary veins	Normal pulmonary vasculature

## 3. Discussion

Given the patient′s subacute presentation of recurrent hemoptysis, her incredibly distinct CT findings of cysts with internal solid components, and her perceived refractoriness to an oral course of antifungal treatment, the medical team was highly suspicious for pulmonary aspergillomas. The most common manifestation of pulmonary aspergillosis is hemoptysis, typically from bronchial vessels as microorganisms directly invade local vessels or release endotoxins that irritate the vasculature lining the cystic cavity [2]. While large‐volume hemoptysis in this setting certainly has lethal capacity, the majority of pulmonary aspergillomas remain stable while a reported 10% spontaneously decrease in size or self‐resolve [3]. As there is no reliable indicator, be it radiographic or clinical, to predict the progression and severity of this pathology, it can be unclear when to opt for treatment. While medical and procedural treatment options exist, their efficacy is often mixed and inconsistent. Although an empiric trial of oral antifungal such as itraconazole or posaconazole offers a low‐risk approach to treatment, studies report a pulmonary aspergilloma cure rate of less than 20% [4]. In life‐threatening cases of hemorrhages, BAE can temporize a patient; however, hemoptysis often recurs due to the development of collateral vasculature [5]. Surgical management offers a more definitive approach to treatment although it is often morbid and exposes patients to risks such as hemorrhage, bronchopleural fistula, and empyema [2].

In this patient′s case, an intermediate option of intracavitary amphotericin was posed prior to considering surgery. This technique, first described in the 1980s, was intended for symptomatic patients whose comorbidities precluded a safe surgical approach [6]. An indwelling percutaneous catheter is placed and amphotericin is directly injected into the cavity [7]. A 2013 trial successfully employed intracavitary amphotericin instillation in 20 patients; 17 of the 20 had resolution of their hemoptysis by discharge, yet 6 of these had recurrence of hemoptysis at their 1‐month follow‐up [8]. As can be expected, the placement of a tunneled catheter into a pulmonary cavitary in this work is not without complication and resulted in pneumothorax in 26% of trialed cases. Given the paucity of long‐term outcome data with this approach, intracavitary instillation of amphotericin remains on the menu of treatment options yet without strong indication.

In our patient′s case, she was presented with an array of procedural options prior to determining next steps. On the menu of IR‐driven interventions, both intracavitary amphotericin and BAE were considered. As intracavitary amphotericin lacks long‐term outcome data, requires close follow‐up, and has tunneled pleural catheter–associated iatrogenesis, the patient was understandably not keen to proceed with this option. Additionally, the uncertainty in a diagnosis of pulmonary aspergilloma further decreased the desiredness of this procedure as this patient may have been exposed to all the risks in treating a presumed fungal etiology that was ultimately proven baseless. BAE also seemed to be a reasonable option but is often reserved for moderate to massive hemoptysis whereas this patient had several occurrences of self‐contained, mild hemoptysis which is defined as hemoptysis less than 100 mL per day or less than 50 mL per episode [9]. In a large systematic review, BAE is also seen to be effective up front in combating hemoptysis but has a high recurrence rate ranging broadly from 10% to 57% in part due to collateral blood flow, incomplete embolization, and other procedural factors [9]. BAE‐associated iatrogenesis is rare but includes devastating complications such as vascular dissection, perforation, TIA, and stroke. Once more, our patient declined this IR‐guided option as she desired both definitive diagnosis and management; she was tired of presenting to the hospital for hemoptysis and wanted to maximize her chance of diagnosis. Therefore, she opted for a surgical approach, namely, a left lower lobe segmentectomy. Regnard et al. describe their series of 89 surgical resections of pulmonary aspergillomas where none of the patients had recurrence of hemoptysis postoperation [10]. Although there were five postoperative deaths, the authors report no death or major surgical complication in asymptomatic patients without underlying pulmonary disease.

While surgery remained the most invasive option for management, our patient preferred to incur more up‐front risk if that allowed for the highest chance of diagnosis and lowest chance of recurrent hemoptysis. She underwent a left lower lobe segmentectomy, ultimately revealing the surprise diagnosis of ILS, a pathology characterized by segments of nonfunctional lung parenchyma that enjoys their own aberrant blood supply and often manifests as hemoptysis as a result [11]. While several other cases have been described of diagnosis of ILS in adulthood [12–14], it has been reported that the majority of cases are diagnosed prior to age 20 and are very rarely diagnosed in adults older than 40 [15]; to our knowledge, this case vignette represents the oldest reported patient with new diagnosis of this congenital anomaly. Prior cases similarly describe the often elusive nature of this diagnosis; patients presenting with recurrent pneumonias, hemoptysis, cough, and chest pain often receive a definitive diagnosis far removed from their initial complaint [16, 17]. Characteristic cross‐sectional imaging findings include a sequestered lung segment with cystic areas of cavitation and surrounding emphysematous lung changes on the lesion′s border [16]; it has been reported that up to 95% of cases involve the lower lung lobes [18]. As witnessed in this case, these imaging findings of a solid mass with or without associated cystic lesions can often masquerade as presumed fungal or rheumatologic pulmonary disease.

As patients have been described as presenting with subsequent complications including recurrent infections and development of an abscess, the therapeutic surgical management often pursued to minimize and mitigate further complications is often what leads to the unexpected diagnosis of ILS on tissue pathology. Prior cases have reported the initial percutaneous embolization of the aberrant vasculature followed by staged surgical resection with standard treatment typically constituting complete lobar resection [14]. Other series proceed operatively without initial embolization; no complications including excessive bleeding were noted in this description of three surgical cases of lobar resection of later diagnosed ILS [15]. Literature review appears to suggest that lobar resection is often the initial therapeutic approach for ILS and a well‐tolerated procedure with the caveat that ILS is often diagnosed in a young and relatively healthy population.

Overall, this case represented a brief opportunity to review pulmonary aspergilloma management but more importantly underscores the importance of maintaining a broad differential and reassessing a diagnosis. As this patient was significantly older than the expected age of diagnosis of the described congenital pathology, ILS and other congenital malformations were deemed to be exceedingly unlikely as the cause of her chronic hemoptysis. Had the medical team continued to tunnel on a diagnosis of pulmonary aspergilloma and opted for the intermediate risk option of intracavitary amphotericin as opposed to surgery, in hindsight the patient would have been exposed to unnecessary and unjustified risk.

As this case appears to represent the oldest patient newly diagnosed with ILS, it serves as a reminder to maintain a high index of suspicion when considering “characteristic imaging findings”; although the cystic lesions with a solid component were quite convincing for fungal etiology, this patient proved to be an exception to Occam′s razor. Given the initial mycotic suspicion, this patient′s case raised several questions related to the medical versus surgical treatment of pulmonary aspergillomas as described above. Further, given this patient′s advanced age and limited functional status, this case further invokes a question of surveillance versus surgical intervention. Although ILS is often fraught with infectious complications, this patient′s only concerns were a few instances of minor hemoptysis over a several month period; as she lived asymptomatically for 84 years prior to identification of this congenital lesion, it is certainly possible that this abnormality left to its own devices would not have represented this patient′s life‐limiting illness. However, given the uncertainty in diagnosis, tissue diagnosis was likely prudent prior to confining this patient to prolonged antifungal treatment for her initially presumed mycotic infection. Finally, given the paucity of cases of ILS, it is difficult to conceive a robust enough cohort of patients to investigate the varying success and complication rates of lobar, sublobar, and staged surgical resection with percutaneous embolization of the aberrant vessel; however, these questions are raised given this patient′s advanced age and comorbidity.

In conclusion, this exceedingly rare case of new diagnosis of ILS in an 84‐year‐old patient further reinforces the adage in clinical medicine that patient pathology is not confined to age or demographic ranges described in the medical texts that we reference.

## Funding

No funding was received for this manuscript.

## Conflicts of Interest

The authors declare no conflicts of interest.

## Data Availability

Data sharing is not applicable to this article as no datasets were generated or analyzed during the current study.
